# Ecofriendly synthesis and characterization of oxygen-enriched g-C_3_N_4_ from diverse precursors for efficient organic dye decontamination

**DOI:** 10.55730/1300-0527.3724

**Published:** 2025-03-01

**Authors:** Muchammad TAMYIZ

**Affiliations:** Department of Environmental Engineering, Faculty of Engineering, Nahdlatul Ulama University of Sidoarjo, Sidoarjo, Indonesia

**Keywords:** Adsorption kinetics, neutral red dye removal, oxygen-doped g-C_3_N_4_, photocatalytic degradation, visible light photocatalysis

## Abstract

Industrial wastewater from sectors such as textiles, printing, and pharmaceuticals contain harmful pollutants, including nonbiodegradable dyes, which pose significant challenges for environmental safety. Neutral red, a cationic dye commonly found in wastewater, obstructs photosynthesis in aquatic ecosystems and carries potential toxicity. Traditional methods of dye removal often prove ineffective due to the chemical stability of these compounds. In this study, oxygen-doped graphitic carbon nitride (O-doped g-C_3_N_4_) was synthesized as an innovative photocatalyst for the degradation of neutral red dye under visible light. The material was synthesized through a sustainable process involving the calcination of urea, dicyandiamide, and oxalic acid, and its characteristics were evaluated using various techniques, including XRD, FT-IR, UV-Vis spectroscopy, and SEM. Photocatalytic degradation of neutral red was analysed using a custom photoreactor under visible light. The results demonstrated that O-doped g-C_3_N_4_ exhibited enhanced photocatalytic efficiency compared to pure g-C_3_N_4_, reducing the recombination of electron-hole pairs and effectively degrading the dye. Adsorption kinetics followed a pseudo-2nd-order model, while adsorption isotherms suggested that the Langmuir model best described the adsorption process, indicating monolayer adsorption. The maximum adsorption capacity of O-doped g-C_3_N_4_ for neutral red was 9.643 mg g^−1^, surpassing pure g-C_3_N_4_. The photocatalytic performance of OCN-UD was assessed under visible light, revealing a significant degradation efficiency of 86% for neutral red after 60 min, compared to 51% for pure g-C_3_N_4_. Kinetic studies indicated that the adsorption of neutral red onto OCN-UD primarily followed a pseudo-2nd-order model, demonstrating chemical adsorption processes. The synergistic effects of adsorption and photocatalysis were evident, as the initial adsorption phase concentrated dye molecules near active sites, facilitating efficient photocatalytic degradation through reactive oxygen species generation. This study highlights the potential of O-doped g-C_3_N_4_ as an efficient, eco-friendly solution for the treatment of dye-laden wastewater.

## 1. Introduction

Daily, industrial wastewater containing a range of pollutants, such as those from the printing, textiles, papermaking, cosmetics, and pharmaceutical industries, as well as organic dyes, is discharged into both surface and groundwater sources. [1–3]. Textile dyes vary widely, are heavily consumed, and possess complex structures along with notable chemical stability, making their biodegradation in water a challenging task [2]. Textile wastewater releases close to 1000 t of nonbiodegradable dyes into natural water sources on an annual basis [4]. A large number of dyes dissolve easily in water, which allows them to enter the environment alongside industrial waste [5]. The processes of oxidation and hydrolysis of organic dyes lead to the formation of more harmful intermediates that can impair the photosynthesis carried out by organisms living in water [6]. Furthermore, several dyes are identified as having harmful, mutagenic, and cancer-inducing effects [7–9].

Neutral red, a cationic dye with an aromatic heterocyclic structure, is seen as harmful but has low toxicity. It is poorly biodegradable and capable of creating deep, vivid colors. Wastewater containing neutral red obstructs light penetration, which hinders photosynthesis in aquatic vegetation. Additionally, neutral red wastewater can negatively affect microbial populations, leading to severe damage to the aquatic ecosystem [8–10]. When subjected to heat, neutral red undergoes thermal decomposition, resulting in the formation of dangerous compounds. Additional harmful effects include its potential to cause cancer, as well as its acute and chronic toxicity [7,8]. Numerous dyes are known for their chemical stability and resistance to degradation, making traditional physical and biological treatment methods inadequate for complete removal [11]. Notably, the efficient treatment of complex organic dyes, both cationic and anionic remains a key focus of international research efforts.

Considered a highly effective method for treating water, semiconductor photocatalysis employs common photocatalysts such as ZnO, TiO_2_, CdS, WO_3_, metal oxide, and ZnS [12]. These semiconductors have the drawback that ZnO becomes unstable in water. Furthermore, CdS, ZnS, and iron oxides may suffer corrosion from photoanodes, and WO_3_ has low levels of photoactivity [4]. Although TiO_2_ continues to be the most widely studied semiconductor due to its effective photocatalytic activity under UV light, ultraviolet radiation constitutes just 2%–3% of sunlight, which considerably limits its application in natural light conditions for environmental restoration [1]. Consequently, there is a pressing need for the development of catalysts that function under visible light.

Graphitic carbon nitride (g-C_3_N_4_) has garnered significant interest as an effective polymeric organic photocatalysts that generate energy and decompose organic pollutants when activated by visible light. This is attributed to its ideal band gap of 2.7 eV, outstanding thermal and chemical stability, scalability for mass production, non-toxic nature, and long-lasting durability [1,13]. Nonetheless, the band gap of pure g-C_3_N_4_ remains relatively large, which limits its efficient use of visible light. Additionally, similar to many single-component photocatalysts, in pure g-C_3_N_4_, there is a high tendency for recombination between the electrons and holes generated by light [14]. Various approaches have been adopted to enhance the photocatalytic efficiency of g-C_3_N_4_ in order to resolve these issues, including developing mesostructured g-C_3_N_4_, integrating it with other materials, or introducing heteroatoms through doping. These modifications have led to improved photocatalytic efficiency relative to pure g-C_3_N_4_ [12,15,16]. Heteroatom doping is considered one of the most promising techniques among the various modification methods.

Introducing heteroatoms into g-C_3_N_4_ has been proven to alter its electron and band gap structure, significantly improving catalytic efficiency. Heteroatom doping, including elements like boron, fluorine, phosphorus, and sulfur, is a well-known approach to modify the surface electronic characteristics of g-C_3_N_4_, thus enhancing its catalytic function [17–22]. Oxygen is frequently considered a preferred dopant due to its eco-friendly properties and oxalic acid is often used as a precursor oxygen doped g-C_3_N_4_. Nevertheless, research on oxygen-doped g-C_3_N_4_ (O-doped g-C_3_N_4_, OCN) remains limited. Some research still relies on a single precursor and oxygen source to generate O-doped g-C_3_N_4_, including combinations like urea combined with hydrogen peroxide, melamine, and ascorbic acid [4,23]. In this study, I unveiled a straightforward and sustainable synthesis approach for O-doped g-C_3_N_4_ (OCN) by calcining ground mixtures of urea, dicyandiamide, and oxalic acid. The abbreviation “OCN-UD” refers to the O-doped g-C_3_N_4_ synthesized using urea-dicyandiamide as a precursor.

The primary goal of this study is to explore the potential of oxygen-doped graphitic carbon nitride (OCN-UD) as an effective photocatalyst for the removal of neutral red dye from wastewater under visible light. Specifically, the author aims to investigate how oxygen doping influences the structural, optical, and photocatalytic properties of g-C_3_N_4_, with a focus on improving its efficiency for dye degradation. Through a detailed comparison with pure CN-UD, this study will assess the impact of oxygen doping on photocatalytic performance, adsorption capacity, and overall degradation efficiency of neutral red dye.

## 2. Materials and methods

### 2.1. Material

In this research, all chemical reagents were of analytical grade, and deionized water (DI) was used throughout the experiments. The chemicals involved included urea (CH_4_N_2_O) 99%, dicyandiamide (C_2_H_4_N_4_) 99%, oxalic acid (C_2_H_2_O_4_) 99%, neutral red (C_15_H_17_C_l_N_4_) 98%, ethanol (C_2_H_6_O) ≥ 99.8%, acetone (CH_3_COCH_3_) ≥ 99%, methanol (CH_3_OH) ≥ 99.9%, isopropyl alcohol (C_3_H_8_O) ≥ 98.5%, sodium hydroxide (NaOH) ≥ 99%, and hydrochloric acid (HCl) 37%, each of which was of analytical grade.

### 2.2. Fabrication of pure g-C_3_N_4_-UD

The pure g-C_3_N_4_-UD (CN-UD) was produced via thermal polycondensation. First, 4 g of urea and 2 g of dicyandiamide were ground into a smooth powder. The powder was placed in a covered crucible for calcination at 550 °C over 4 h, with a heating rate of 4 °C per min, and allowed to cool down naturally. The resulting yellow powder was reground, washed with deionized water and ethanol three times, and then dried.

### 2.3. Synthesis of O doped g-C_3_N_4_-UD (OCN-UD)

The preparation of O doped g-C_3_N_4_ involved mixing 2 g of dicyandiamide, 4 g of urea, and 0.6 g of oxalic acid, followed by grinding the mixture in an agate mortar for 30 min. The powder was then placed in a tube muffle furnace at 550 °C for 4 h in an air atmosphere. Once the sample cooled naturally to room temperature, it was ground again in an agate mortar.

### 2.4. Characterization of OCN-UD

The morphology and microstructure were analyzed using Scanning Electron Microscopy (SEM) and the crystal structure was examined through the application of X-ray Diffraction (XRD) with a wavelength of 1.5406 Å over a 2θ range of 10° to 80°. UV-Vis spectrophotometry was used to record the absorption spectrum from 350 to 800 nm, and to identify the functional groups of the photocatalyst, Fourier-transform infrared (FT-IR) spectroscopy was conducted utilizing the KBr method. The photoluminescence (PL) spectra were acquired using a Hitachi F-7000 fluorescence spectrophotometer equipped with a 150 W Xenon lamp, utilizing 375 nm as the excitation wavelength.

### 2.5. Photocatalytic degradation test

A custom photoreactor was employed to assess the photocatalytic degradation of neutral red. Initially, 10 mg of the photocatalyst was precisely weighed. A solution containing 10 mL of neutral red at a concentration of 10 mg L^−1^ was made. Before light irradiation, the mixture was stirred in darkness for 30 min to establish adsorption equilibrium. The solution was then exposed to eight 8 W LED visible light lamps at a wavelength of 460 nm, with 1 mL samples collected at ten-min intervals. After being centrifuged for 5 min, the samples were filtered using a PTFE membrane with a pore size of 0.22 μm. Analysis of the remaining residue was conducted with a B-One UV-Vis spectrophotometer, while the maximum wavelength of neutral red, the targeted pollutant, was 584 nm. Moreover, the pH was modified by incorporating 0.1 M HCl and 0.1 M NaOH into the solution.

### 2.6. Model of adsorption kinetics and isotherm

Various factors impact the kinetics of adsorption, including the reaction order, capacity for adsorption, and the speed at which adsorption occurs. In order to characterize the dynamics of neutral red adsorption on O doped g-C_3_N_4_, the kinetic models of pseudo-1st-order (PFO) and pseudo-2nd-order (PSO) reactions were employed. Moreover, the adsorption isotherm model elucidates the distribution of neutral red molecules at the liquid-solid interface during equilibrium, indicating whether this distribution is homogeneous or heterogeneous. It also reveals details about the active sites and whether adsorption occurs in a monolayer or multilayer format.

## 3. Results and discussion

**Figure 1:**
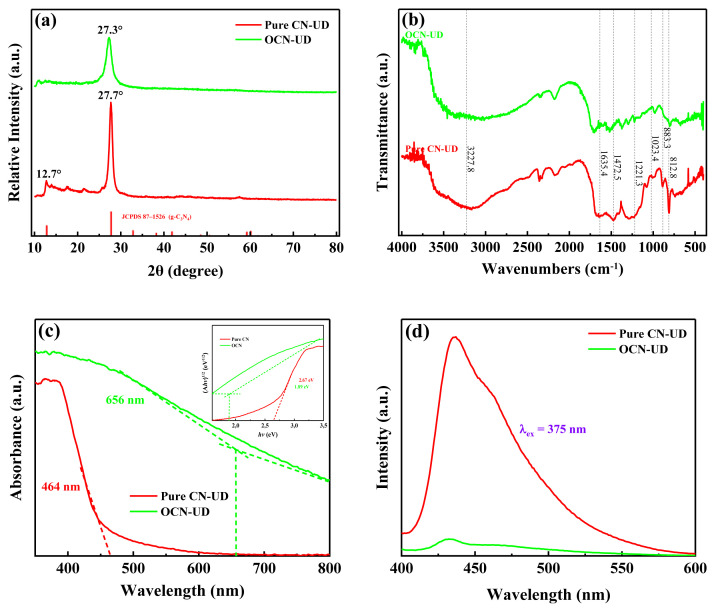
(a) X-ray diffraction pattern, (b) FT-IR spectra, (c) Tauc plot, and (d) PL spectra of pure CN-UD and OCN-UD.

Figure 1a displays the XRD patterns of pure CN-UD and OCN-UD samples. The pure CN-UD sample displays two peaks: a weak peak at 12.7° corresponding to the (100) plane, attributed to the in-plane repetition of triazine rings, and a strong peak at 27.7° related to the (002) planes of hexagonal g-C_3_N_4_, which reflects the interlayer characteristics of its graphitic structure [13,14,16,24]. The introduction of oxalic acid caused the diffraction peak of the (002) lattice plane to shift to a lower angle of 27.22°, which indicates an increase in the interlayer spacing of OCN-UD due to the incorporation of oxygen atoms [25,26]. Concurrently, the broadening of the diffraction peak for the (100) lattice plane implies that the in-plane layer dimensions of OCN-UD were reduced, this reflects the effective doping of oxygen within the g-C_3_N_4_ framework.

The FT-IR spectra of the samples are presented in Figure 1b. In the spectrum for pure CN-UD, a strong peak is observed at 812.8 cm^−1^ and a weaker one at 883.3 cm^−1^; these features correspond to the out-of-plane breathing modes of heptazine rings and the N–H distortion mode found in pure CN-UD, respectively [22,27,28]. The peaks found between 1230.4 and 1635.4 cm^−1^ correspond to the vibrational nodes associated with the stretching of the repeating units lacking heptazine (C-N heterocycles) [29]. The stretching vibrations of the N-H and O-H groups, characterized by a broad band, may play a role in the additional band observed at 3227.8 cm^−1^ within the OCN-UD structure [28]. Generally, the weak peak observed at 1023.4 cm^−1^ in OCN-UD corresponds to the stretching vibration of C–O bond, but this peak appears very faint in the OCN-UD samples. Moreover, Characteristic peaks of the tri-s-triazine unit are clearly evident in the FT-IR spectra of the samples, suggesting that O doping retains the core structure of g-C_3_N_4_. Relative to CN-UD, the absorption peaks at 1221.3 and 1463.1 cm^−1^ in OCN-UD decrease by 9 cm^−1^, probably due to the high electronegativity of the incorporated oxygen, which affects the charge distribution surrounding the C=N and C-N bonds.

Optical properties of the prepared photocatalyst were investigated using UV–Vis DRS spectroscopy. As depicted in Figure 1c, the absorption edge of the OCN-UD samples shifted to a longer wavelength of 656 nm, in contrast to pure CN-UD, which has an absorption edge at 464 nm. Considering that both CN-UD and its modified version (OCN-UD) are direct band gap semiconductors, the Tauc function was plotted against photon energy (hν) to determine the band gap energies of pure CN-UD and OCN-UD samples [28,30,31]. As indicated in the inset of Figure 1c, there is a reduction in the band gap energy for the OCN-UD samples (1.89 eV) relative to pure CN-UD (2.67 eV), consistent with prior findings [31].

The photoluminescence (PL) spectra depicted in Figure 1d show that pure CN-UD has a higher luminescent intensity than OCN-UD, suggesting that the process of oxygen doping lowers the photo-generated electrons and holes recombination rate. This doping in g-C_3_N_4_ introduces electron-trapping centers and alters the conduction band, effectively separating photogenerated charge carriers. The OCN-UD displays the lowest luminescence, indicating the most reduced recombination rate [32,33]. In CN-UD, the electronic band states consist of the σ band derived from sp^3^ C–N bonds, the π band originating from sp^2^ C–N bonds, and a lone pair (LP) state associated with the nitrogen atom acting as a bridge in the structure [4].

**Figure 2:**
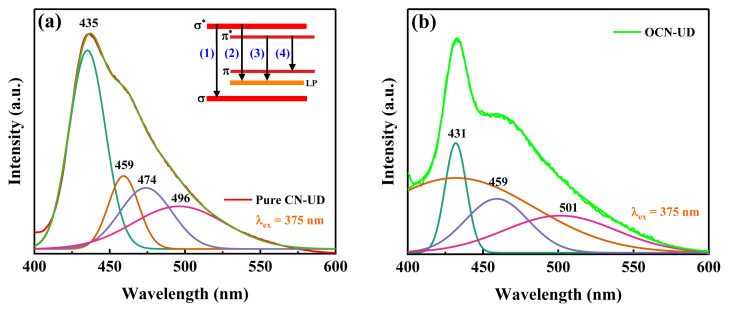
The deconvoluted photoluminescence spectra of (a) CN-UD and (b) OCN-UD.

As shown in the inset of Figure 2a, CN-UD exhibits four distinct peaks, representing various electron transitions. The transitions consist of four types: (1) from the σ* state in the conduction band to the σ state in the valence band, (2) from the σ* state in the conduction band to the LP state, (3) from the π* state in the conduction band to the LP state, and (4) from the π* state to the π state in the valence band [31,34,35]. Figures. 2a and b present the resolved spectra of CN-UD and OCN-UD, showing peaks at 435 nm (2.85 eV), 459 nm (2.70 eV), 474 nm (2.62 eV), and 496 nm (2.50 eV) for CN-UD, whereas OCN-UD peaks are observed at 431 nm (2.88 eV), 459 nm (2.70 eV), and 501 nm (2.48 eV). The blue shift in the first three OCN-UD peaks compared to pure CN-UD indicates increased emission energy, correlating with the smaller band gap of OCN-UD. In contrast, the red shift observed in the fourth peak is appropriate to a smaller energy gap between the π and π* bands in Oxygen-doped g-C_3_N_4_-UD compared to g-C_3_N_4_-UD [36].

**Figure 3:**
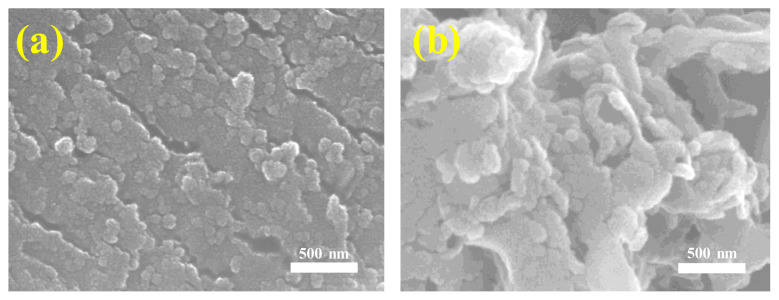
SEM images of (a) CN-UD and (b) OCN-UD.

Figures 3a and 3b present the SEM images of the pure CN-UD and OCN-UD samples. Pure CN-UD displays flake-like or thick sheet-like structures, along with irregular forms and a multi-layered block configuration, typically measuring several hundred nanometers in size. In contrast, the OCN-UD samples exhibit a porous structure that is irregular and fluffy, consisting of fragmented and coiled nanosheets [15]. This finding indicates that the use of various precursors—specifically urea, dicyandiamide, and oxalic acid—altered the interaction forces among precursor molecules during thermal polymerization, resulting in significant changes in morphology.

### 3.1. Neutral red degradation through synergistic adsorption-photocatalysis

**Figure 4:**
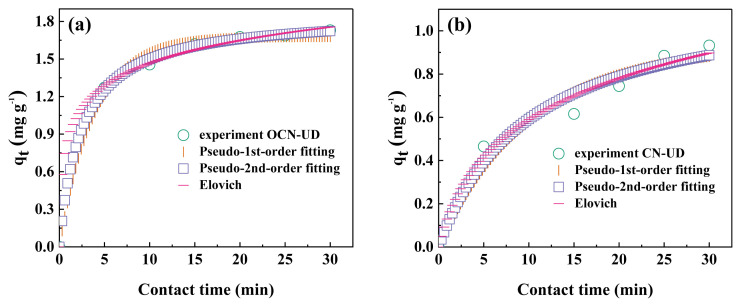
The nonlinear regression of pseudo-1st-order, pseudo-2nd-order, and Elovich models of (a) OCN-UD and (b) CN-UD.

Several kinetic models, including pseudo-1st-order, pseudo-2nd-order, and Elovich, were applied to assess the adsorption kinetics. The nonlinear regression analyses for the pseudo-1st-order, pseudo-2nd-order, and Elovich models are presented in Figures 4a and 4b. Table 1 summarizes the equations and parameters obtained for the adsorption of neutral red dyes. The pseudo-2nd-order model, with R^2^ values of 0.967 and 0.999 for pure CN-UD and OCN-UD, respectively, provided the best fit, indicating that neutral red adsorption adheres to this model. This points to chemical adsorption driven by van der Waals interactions, where electron sharing or exchange occurs between the adsorbent and neutral red molecules [23].

The Elovich model also shows good agreement with the kinetic data, with R^2^ values of 0.977 and 0.998 for pure CN-UD and OCN-UD, indicating the presence of chemical reactions taking place on heterogeneous surfaces. Significantly, the increased α and β values in the Elovich model for OCN-UD indicate a quicker adsorption rate of neutral red compared to pure CN-UD. As a viable approach for removing organic dye contaminants from water, adsorption is impacted by variables including the initial concentration of neutral red dyes.

This study of isotherms aims to analyze the effect of the initial concentration of neutral red dyes on adsorption capacity, focusing on distinguishing between physical and chemical adsorption and exploring how different adsorbent surfaces affect the uptake of neutral red molecules. As a result, the Langmuir, Freundlich, and Temkin isotherm models were applied in the isotherm study to examine the adsorption of neutral red by CN-UD and OCN-UD adsorbents.

**Figure 5:**
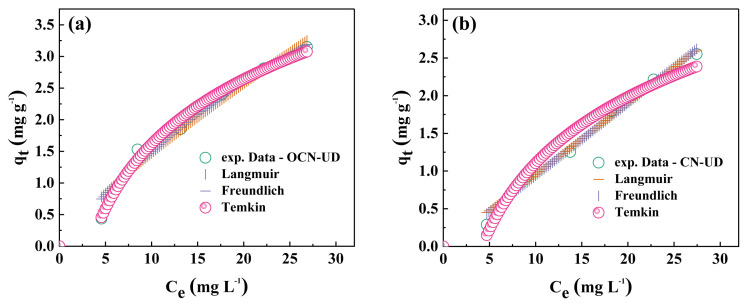
The Langmuir, Freundlich, and Temkin isotherm models of (a) OCN-UD and (b) CN-UD.

Figures 5a and 5b display the nonlinear regression analysis of isotherm models for the adsorption process using OCN-UD and CN-UD adsorbents, while Table 2 provides the corresponding model parameters. For the Langmuir model, the correlation coefficients (R^2^) in the adsorption of neutral red by CN-UD and OCN-UD were 0.988 and 0.968, respectively, indicating a better fit compared to the Freundlich isotherm model. These strong correlations indicate that the experimental data align closely with the Langmuir model, pointing to monolayer adsorption on a homogeneous surface that contains a fixed number of equivalent adsorption sites with neutral red molecules.

The adsorption capacities of neutral red on CN-UD and OCN-UD were found to be 4.405 mg g^−1^ and 9.643 mg g^−1^, respectively, indicating that the modification of CN-UD significantly improved its capacity and efficiency. The R^2^ values for both the isotherm between Langmuir and Freundlich models did not show significant variation, suggesting that the adsorption process involves both homogeneous and heterogeneous surfaces. Furthermore, values of n ≈ 1and E > 8 kJ mol^−1^ validate that neutral red adsorption is favorable and is largely governed by chemisorption. Moreover, the b*_T_* parameter for neutral red adsorption based on the Temkin model was 1953 kJ mol^−1^ for CN-UD and 1678 kJ mol^−1^ for OCN-UD, indicating a chemical and endothermic adsorption process driven by chemical binding or electrostatic forces [37]. The parameter b*_T_* is related to the heat of adsorption and represents the energy variation due to adsorbate interactions with the adsorbent surface. The Temkin model assumes that the heat of adsorption decreases linearly with coverage. While, the unit of b*_T_* is typically in kJ mol^−11^, which reflects the energy per mole of the adsorbate molecules.

**Figure 6:**
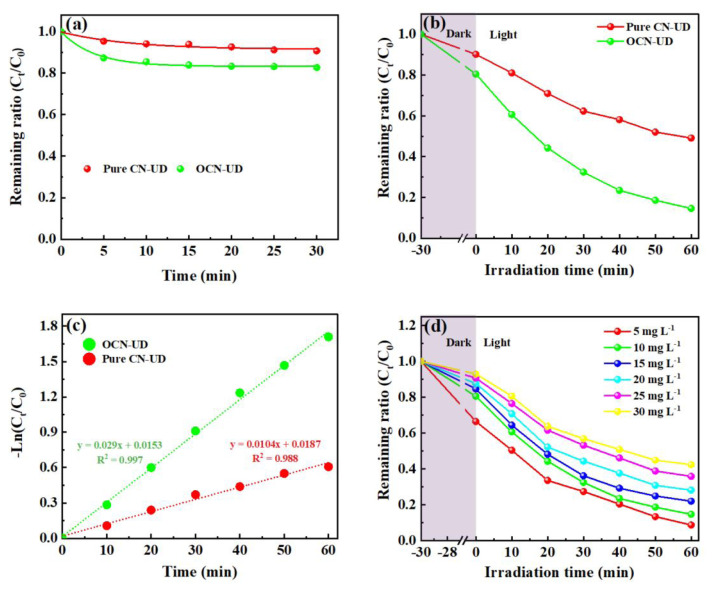
(a) Adsorption equilibrium of CN-UD and OCN-UD, (b) photodegradation of neutral red over of CN-UD and OCN-UD, (c) the reaction rate constants of CN-UD and OCN-UD, and (d) the effects of initial neutral red concentration.

This study explored the synergistic effects of adsorption and photocatalytic degradation of neutral red using CN-UD and OCN-UD photocatalysts in both illuminated and dark environments. The main purpose was to assess adsorption’s contribution by performing experiments in darkness, thus excluding photocatalysis. This ensured that the results reflected adsorption as the only mechanism responsible for neutral red removal from the solution. Adsorption experiments were carried out with a 10 mg L^−1^ neutral red solution at a neutral pH of 7, using 1.0 g L^−1^ of the synthesized OCN-UD samples. These conditions were selected to concentrate on adsorption without interference from photocatalytic reactions. The observations showed that equilibrium was attained in 30 min, with the bulk of adsorption happening within the initial 10 min, then transitioning into a slower phase. The OCN-UD showed a maximum adsorption capacity of 1.863 mg g^−1^, notably higher than the 1.182 mg g^−1^ capacity of CN-UD. The OCN-UD removed 17% of neutral red compared to the 9% removal by CN-UD (Figure 6a), indicating that oxygen doping enhanced adsorption performance, likely by increasing the surface area or introducing new active sites for adsorption [12].

The swift adsorption observed in the first 10 min demonstrates OCN-UD’s strong affinity for neutral red, with an adsorption capacity of 1.55 mg g^−1^ reached quickly. After this fast phase, the rate of adsorption slowed down, reaching equilibrium by 30 min, indicating that no more adsorption occurred. This behavior is characteristic of adsorption systems, where a swift initial phase transitions into a slower phase toward equilibrium as the adsorbent’s surface sites reach saturation. Moreover, the close alignment between the pseudo-2nd-order model and experimental outcomes suggests that neutral red adsorption onto OCN-UD is primarily a chemically controlled process [20]. The mesoporous structure of the material backs this finding, as it improves the diffusion of adsorbate molecules and promotes an environment conducive to chemical interactions. In the next experiment, pure CN-UD and OCN-UD were exposed to visible light from an LED lamp to test their degradation efficiency for neutral red. After 60 min, pure CN-UD removed 51% of the dye, while OCN-UD achieved an 86% removal efficiency (Figure 6b). This improvement is credited to the oxygen incorporation in g-C_3_N_4_, which enhances the photocatalyst’s ability to absorb visible light and reduces the recombination rate of electron-hole pairs (e^−^–h^+^ pairs). A lower recombination rate is critical for boosting photocatalytic efficiency by making more electron-hole pairs available for driving pollutant-degrading reactions [12].

The reaction rate constants of the two photocatalysts were used to assess their kinetic behavior. For pure CN-UD, the rate constant was 0.0104 min^−1^, while for OCN-UD, it was significantly higher at 0.0290 min^−1^ (Figure 6c), representing a threefold increase. This improvement in the reaction rate highlights the superior photocatalytic performance of OCN-UD, which can degrade neutral red much faster, making it more suitable for large-scale environmental applications like water purification. In the case of OCN-UD, the rate constant for adsorption was found to be 0.005 min^−1^, while the rate constant for photodegradation was 0.0290 min^−1^. The observed improvement in the photodegradation rate in the presence of adsorption indicates a synergistic effect, where the combination of adsorption and photocatalytic degradation leads to a more efficient overall process. This supports the synergistic nature of the coupling between these two processes. Figure 6d displays that the initial concentration affects the photodegradation of neutral red by OCN-UD. The efficiency drops from 91% at 5 mg L^−1^ to 58% at 30 mg L^−1^, which is attributed to the limited availability of active sites on OCN-UD. At low concentrations, the photocatalyst’s active sites effectively adsorb and degrade neutral red. However, at higher concentrations, the dye competes for these sites, and degradation intermediates accumulate, occupying the active sites and lowering efficiency.

**Figure 7:**
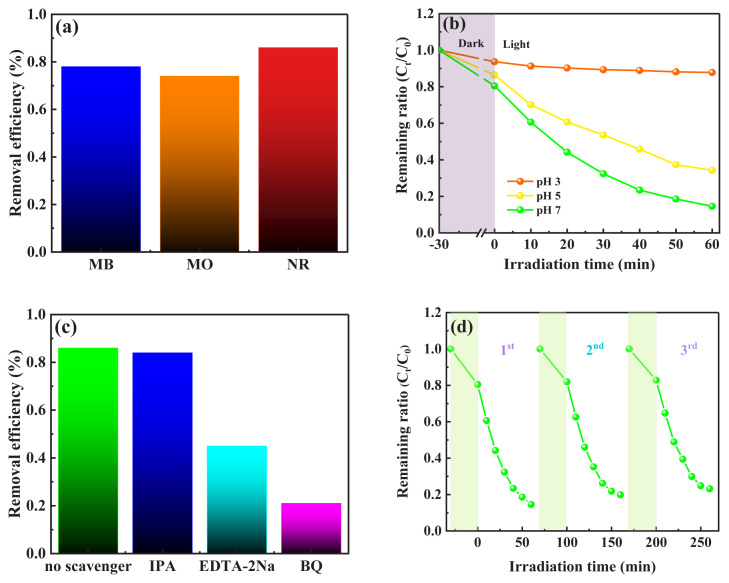
The effect of various (a) organic dye, (b) pH, (c) trapping agent, and (d) recyclability of the OCN-UD in the photodegradation of neutral red under LED visible light irradiation.

Textile industries produce and apply large quantities of dyes each year, making their effective degradation a necessity. To demonstrate the photocatalytic potential of OCN-UD for breaking down various types of dyes, the degradation of several organic dyes was studied under LED visible light irradiation. Figure 7a presents a comparison of the photocatalytic degradation of methylene blue (MB), methyl orange (MO), and neutral red (NR) using OCN-UD over a 60-min irradiation period. The degradation efficiencies for MB, NR, and MO were 78%, 86%, and 74%, respectively. These results show that OCN-UD exhibited different levels of effectiveness in degrading various organic dyes through the photocatalytic process under LED visible light. This variation is mainly due to the differences in the chemical structures of dyes, compound sizes, and electric charges of dyes.

The initial pH of the reaction solution plays a significant role in determining the adsorption and photodegradation efficiency of a photocatalyst for a given target. This is because the pH influences the concentration of hydrogen ions, which can compete with positively charged dye ions for active sites on the photocatalyst. As demonstrated in Figure 7b, the photodegradation of neutral red dye on OCN-UD was evaluated under controlled conditions, such as a neutral red dye concentration of 10 mg L^−1^ and an OCN-UD dose of 10 1 g L^−1^, with pH values ranging from 3 to 7. It was observed that the neutral red dye precipitated when the pH was higher than 7 [38]. The results showed that increasing the pH from 3 to 7 improved the photocatalytic degradation of neutral red, with removal efficiencies of 12% at pH 3, 66% at pH 5, and 86% at pH 7, indicating that pH 7 is optimal for OCN-UD to effectively degrade neutral red. At lower pH values, the decreased degradation efficiency was due to the excess hydronium ions (H^+^) competing with the neutral red dye for the available sorption sites on the photocatalyst surface. Additionally, since neutral red is a cationic dye, it exists as positively charged ions in solution. The point of zero charge (pzc) for OCN-UD is well-known, showing that OCN-UD surfaces are positively charged in acidic conditions (pH < 7) and negatively charged in alkaline conditions (pH > 7) [39]. This causes a repulsive interaction between the positive surface charge of OCN-UD and the cationic neutral red dye, leading to reduced photodegradation efficiency [38]. However, at pH 7, where OCN-UD has a negative charge, π–π and electronic interactions between neutral red molecules and OCN enhance the degradation process.

To gain a deeper understanding of the enhanced photodegradation mechanism of OCN-UD on neutral red molecules, the active species involved in the process were investigated. As shown in Figure 7c, the photocatalytic degradation of neutral red by OCN-UD was evaluated in the presence of several scavengers, such as disodium ethylenediamine tetraacetic acid (EDTA-2Na), which traps photogenerated holes (h^+^), p-benzoquinone (BQ), which scavenges superoxide radicals (^•^O_2_^−^), and isopropanol (IPA), which captures hydroxyl radicals (^•^OH) [40,41]. In the absence of any scavenger, the degradation proceeded at the expected. However, when BQ was introduced, the photocatalytic degradation of neutral red was significantly reduced to just 21%, emphasizing the vital role of ^•^O_2_^−^ in the process. The addition of EDTA-2Na resulted in a decrease in degradation to 45%, suggesting that h^+^ plays a key role in the photocatalytic activity of OCN-UD. Conversely, the presence of IPA caused no significant change in photodegradation, indicating that ^•^OH radicals are not produced during the degradation process. This lack of ^•^OH generation is likely due to the VB potential of OCN-UD being lower than the potential required to generate ^•^OH from H_2_O/^•^OH (1.99 V vs. NHE) [9,14]. Therefore, it can be concluded that ^•^O_2_^−^ and h^+^ are the primary active species driving the photodegradation of neutral red in this system.

Furthermore, the photocatalyst’s recyclability was assessed by testing its performance over multiple reaction cycles. After each cycle, the mixture was centrifuged, and the solid catalyst was washed with methanol and dried at 110 °C for 4 h before being reused in the following cycle. Figure 7d shows that the results from three consecutive runs revealed only a 9% decline in photocatalytic efficiency for neutral red degradation, with removal efficiencies of 86% in the first run and 77% in the third. This indicates that the photocatalyst experienced minimal deactivation over multiple cycles. Deactivation during repeated cycles is commonly observed in carbon-based photocatalysts, primarily due to changes in the physicochemical properties of their surfaces [39]. However, the photocatalytic performance of OCN-UD demonstrates excellent recyclability for neutral red photodegradation.

**Figure 8:**
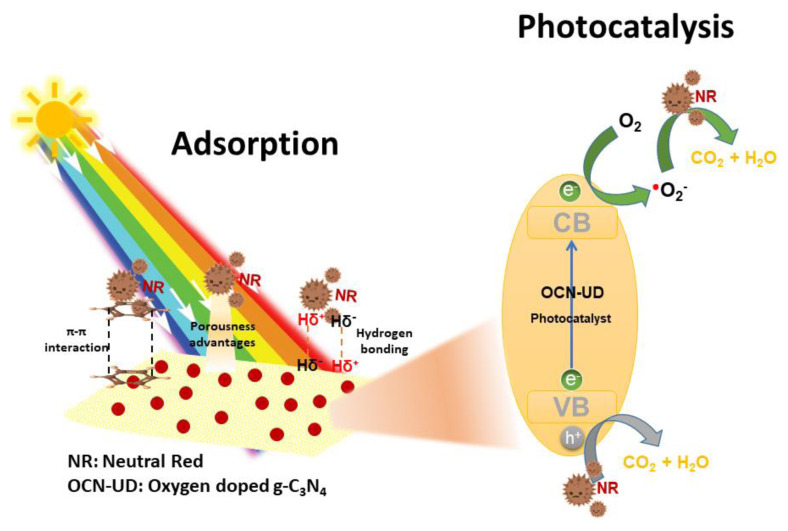
A schematic diagram illustrating the mechanism of photocatalytic degradation of neutral red dye using the OCN-UD photocatalyst.

Figure 8 illustrates the mechanism of neutral red degradation by OCN-UD through two types of reactive oxygen species (ROS). Typically, the photoinduction of OCN-UD occurs when it is exposed to visible light radiation. During this process, photogenerated electrons in the valence band (VB) are transferred to the conduction band (CB), creating an equal number of photogenerated h^+^ in the CB. In the neutral red solution, the oxygen and photogenerated electrons produce ^•^O_2_^−^, while photogenerated h^+^ are also formed. These h^+^ can directly oxidize the neutral red dye, leading to its degradation. Among the two reactive species, ^•^O_2_^−^ radicals play the most significant role in the degradation process, with photogenerated h^+^ playing a secondary role, as demonstrated by scavenger experiments. Both ^•^O_2_^−^ and h^+^ have mineralizing capabilities, breaking down neutral red into smaller molecular substances such as CO_2_ and H_2_O. Furthermore, several factors contribute to the improved photocatalytic performance of OCN-UD in degrading neutral red compared to CN-UD. The oxygen doping adds extra energy levels to the material’s band structure, enabling OCN-UD to absorb more visible light and effectively generate electron-hole pairs [42]. Furthermore, the oxygen atoms act as electron traps, slowing the recombination of electron-hole pairs and extending their lifetime, making them more effective for photocatalytic reactions. In contrast, pure CN-UD exhibits faster recombination rates, which limit its photocatalytic efficiency since the electron-hole pairs do not have enough time to participate in reactions. Oxygen doping resolves this issue, enhancing both light absorption and the material’s photocatalytic efficiency. This combination of improved light absorption, reduced recombination, and optimized structure makes OCN-UD highly effective for environmental pollutant removal.

## 4. Conclusion

In this study, the synthesis of oxygen-doped urea-dicyandiamide-derived g-C_3_N_4_ (OCN-UD) was successfully achieved, and its adsorption and photocatalytic performance were compared with that of pure CN-UD. The results demonstrated that oxygen doping significantly enhanced the photocatalytic degradation efficiency of neutral red dye under visible light. The OCN-UD exhibited an enhanced adsorption capacity (9.643 mg g^−1^) compared to CN-UD (4.405 mg g^−1^), which can be attributed to the increased surface area and the introduction of new active sites. This improvement aligns with previous studies that have shown that doping g-C_3_N_4_ with oxygen can modify its electronic structure, enhance light absorption, and reduce electron-hole recombination. Moreover, the OCN-UD exhibited superior photocatalytic performance, with an 86% degradation efficiency after 60 min, which is notably higher than the 51% removal achieved by pure CN-UD. These findings are consistent with earlier works that reported similar improvements in photocatalytic activity through oxygen doping of g-C_3_N_4_. Additionally, the faster reaction rate constant (0.0290 min^−1^) for OCN-UD further supports the notion that oxygen doping promotes a more efficient photocatalytic process by stabilizing charge carriers, as suggested by other studies on modified g-C_3_N_4_. This demonstrates the potential of OCN-UD as an effective photocatalyst for environmental applications such as water purification, where rapid and efficient removal of organic pollutants is critical. Future research could explore the degradation of other organic dyes and evaluate the performance of OCN-UD in real wastewater conditions to further understand its practical implications.

## Figures and Tables

**Table 1:** The kinetic model result of pure CN-UD and OCN-UD.

Model	Parameter	Adsorbent	
		OCN-UD	CN-UD
Pseudo-1st-order	K_1_ (min^−1^)	0.258	0.099
	q_e_ (mg g^−1^)	1.678	0.928
	R^2^	0.993	0.952
	RMSE	0.047	0.063
Pseudo-2nd-order	K_2_ (g (mg·min)^−1^)	0.221	0.085
	q_e_ (mg g^−1^)	1.863	1.182
	R^2^	0.999	0.967
	RMSE	0.020	0.053
Elovich	α (mg. (g. min)^−1^)	6.977	0.177
	β (g.mg^−1^)	3.807	3.227
	R^2^	0.998	0.977
	RMSE	0.024	0.044

**Table 2:** The isotherm model result of pure CN-UD and OCN-UD.

Model	Parameter	Adsorbent	
		OCN-UD	CN-UD
Langmuir	q_m_ (mg g^−1^)	9.643	4.405
	K_L_ (L mg^−1^)	0.018	0.0002
	R^2^	0.968	0.988
	RMSE	0.159	0.084
Freundlich	K_F_ ((mg g^−1^)/(L mg^−1^)^1/n^)	0.230	0.0896
	N	1.243	0.981
	R^2^	0.959	0.988
	RMSE	0.181	0.084
Temkin	K_T_ (L g^−1^)	0.298	0.239
	b*_T_* (kJ mol^−1^)	1678	1953
	R^2^	0.987	0.965
	RMSE	0.103	0.144
